# Candle Soot Coating for Latent Fingermark Enhancement on Various Surfaces

**DOI:** 10.3390/s17071612

**Published:** 2017-07-11

**Authors:** Qianhui Wei, Yu Zhu, Shouliang Liu, Yongjie Gao, Xiaolong Li, Mi Shi, Xueji Zhang, Meiqin Zhang

**Affiliations:** Research Center for Bioengineering and Sensing Technology, School of Chemistry & Biological Engineering, University of Science & Technology Beijing, Beijing 100083, China; wqhwanwan@126.com (Q.W.); zhuyuiverson@163.com (Y.Z.); liushouliang2006@126.com (S.L.); gaoyongjieustb@126.com (Y.G.); 13269392730@163.com (X.L.); s20160885@xs.ustb.edu.cn (M.S.)

**Keywords:** latent fingermark, candle soot, nanoparticle, visualization

## Abstract

We demonstrate a facile method termed candle soot coating (CSC) for fast developing latent fingermarks (LFMs) on various kinds of surfaces (glass, ceramic, metal, paper and adhesive tape). The CSC method can be considered as simple, fast, and low-cost as well as providing high contrast for LFM visualization in potential forensic applications.

## 1. Introduction

Fingermarks have been recognized as the most powerful evidence of personal identification in forensic investigation for over a hundred years [1,2]. The characteristic patterns on the finger pads and palms are unique to each individual and remain unchanged throughout a person’s lifetime. Latent fingermark (LFM) that is not readily visible to the naked eye refers to the friction ridge pattern left by the fingertip when touching a surface. Therefore, specific treatments are usually required to visualize the LFM pattern for further forensic identification.

Until now, a large number of methods including chemical, physical and optical enhancements have been developed in LFM detection. In the past, powder dusting, cyanoacrylate fuming and ninhydrin dying were the most commonly used techniques of LFM development [3,4,5]. They are effective under most ordinary circumstances, while they are toxic to operators’ health and not always suitable for all the fingermarks encountered. Some other approaches, such as vacuum metal deposition (VMD), multi-metal deposition (MMD) and electrochemical deposition (ECD) are notable techniques for LFM detection on different surfaces, while they also have their own shortcomings [6,7,8,9,10,11,12,13]. VMD requires special vacuum instruments, which limits its wider application. In addition, MMD is very time-consuming with multiple steps and poor repeatability. The drawback of ECD is that it is only suitable for the LFMs on conductive substrates. In recent years, a series of novel techniques has been involved in LFM detection as powerful tools to overcome the sensitivity and selectivity issues currently encountered, including the immuno-labeling technique, electrochemical surface plasmon resonance, the nanoplasmonic method, mass spectrometry, Fourier-transform infrared spectroscopy, Raman spectroscopy, electrochemiluminescence and scanning electrochemical microscopy [14,15,16,17,18,19,20,21,22]. However, most of these methods involved sophisticated or expensive instruments, or labor-consuming protocols. Forensic researchers have always been pursuing the development of novel techniques and reagents which are fast, simple, low-cost, friendly, non-invasive and portable for obtaining high resolution fingermark information for personal identification.

Carbon soot is the dominant solid product of all types of combustion processes and it is used as the source when producing various carbon nanostructures such as carbon black, fullerenes, carbon nanodots, carbon nanotubes, etc. [23,24,25,26]. The carbon soot from the combustion of paraffin wax (candle flame) consists of about 91.69% carbon materials with a small amount of hydrogen, nitrogen, oxygen and other insoluble hydrophobic materials. In 2012, Vollmer et al. characterized the structure and super-hydrophobicity behavior of candle soot film, which was composed of regular carbon nanoparticles (20–50 nm) [27]. Moreover, the carbon nanoparticles in candle soot have excellent optical properties and the structural and better quality of graphitization in the candle soot mean that it has great potential as a material for various applications [28,29,30]. 

A candle flame has three distinct regions: the innermost zone, the middle zone and the outer zone [31]. The inner flame soot particles are superhydrophobic and superoleophilic, because they are relatively large, aggregated particles, and are mostly composed of organics.

Herein, for the first time, we demonstrated an approach that visualized LFMs on various kinds of substrates (glass, ceramic, metal, paper and adhesive tape) by just burning a candle and soot deposition in a fast and simple way.

## 2. Experimental and Discussion

**Figure 1:**
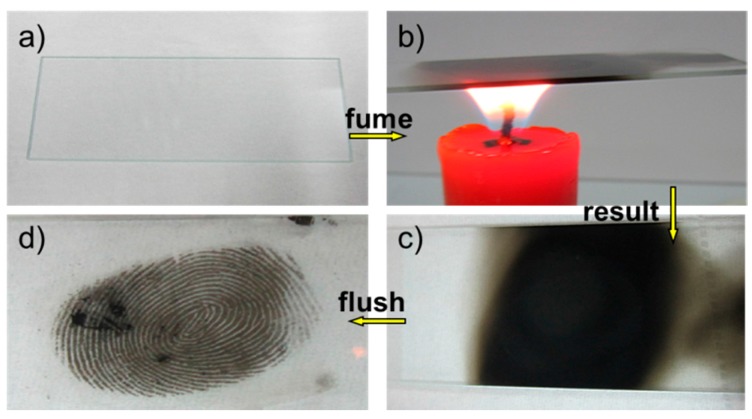
A general protocol of the candle soot coating (CSC) method: (**a**) A latent fingermark (LFM) on a glass slide surface; (**b**) The LFM sample is held above the flame of a candle for soot deposition; (**c**) Fingermark area on the glass slide is fully covered by CSC; (**d**) LFM developed by the CSC method after flushing.

Figure 1 shows a general protocol of LFM visualization on a glass slide surface by a CSC method. First of all, prior to each mark donation, the donor washed his/her hands with mild detergent and dried them on a paper towel thoroughly. LFMs were collected and loaded on the glass slide gently from the same donor after washing his/her hands followed by rubbing the fingertips over the forehead or nose to allow the sebaceous component to predominate in the mark. As can be seen from Figure 1a, the fingermark pattern could hardly be observed at this stage. Secondly, fuming treatment was carried out at the centered flame of a paraffin candle (Figure 1b) for 10–20 s until the glass slide with the fingermark turned black. During this step, the fingermark sample should be pre-heated by a flame back and forth to avoid the glass slide breaking into pieces. When the fingermark region was fully covered by CSC (Figure 1c), the candle was removed and allowed to cool down. After the candle fuming, the fingermark sample was positioned at around 10 cm below the faucet and rinsed with running water whose flow velocity was about 1.5 m/s to flush the excess candle soot and then dried by a gentle N_2_ stream. The candle flame produces carbon nanoparticles on ridge regions and creates super-hydrophobicity. However, the carbon soot nanoparticles are easily peeled off by water flushing due to the weak physical interactions in the nano-agglomerates. The developed LFM, as shown in Figure 1d, could be easily visualized by the naked eye. The LFM components mainly involve a complex mixture of natural secretions of the skin, which is assigned to secretion from two kinds of sweat glands: sebaceous glands found on the facial areas and eccrine glands found on the hands. The sweat secreted from eccrine glands consists of 98–99% water, various inorganic salts, such as chloride, bromide, iodide and phosphate, and of different organic compounds, such as amino and fatty acids, urea etc. [31]. Sebaceous glands excrete sebum fluid, which is mainly composed of fatty acids, glycerides, cholesterol, squalene and a variety of lipid esters [32]. As the relative hydrophobic secretions of the sebaceous LFMs exist, candle soot nanoparticles which are generated from the centered flame are more likely to adhere to the hydrophobic ridges rather than the furrows and the bare glass substrates, which enhanced the spatial pattern of the LFM remarkably. The adhesion of soot on the sebaceous fingermark residues is a complex process but is mainly composed of mechanical adherence, chemical forces and dispersive forces. The chemical/molecular interactions consist of hydrogen bonding, electrostatic interaction and van der Waals force.

**Figure 2:**
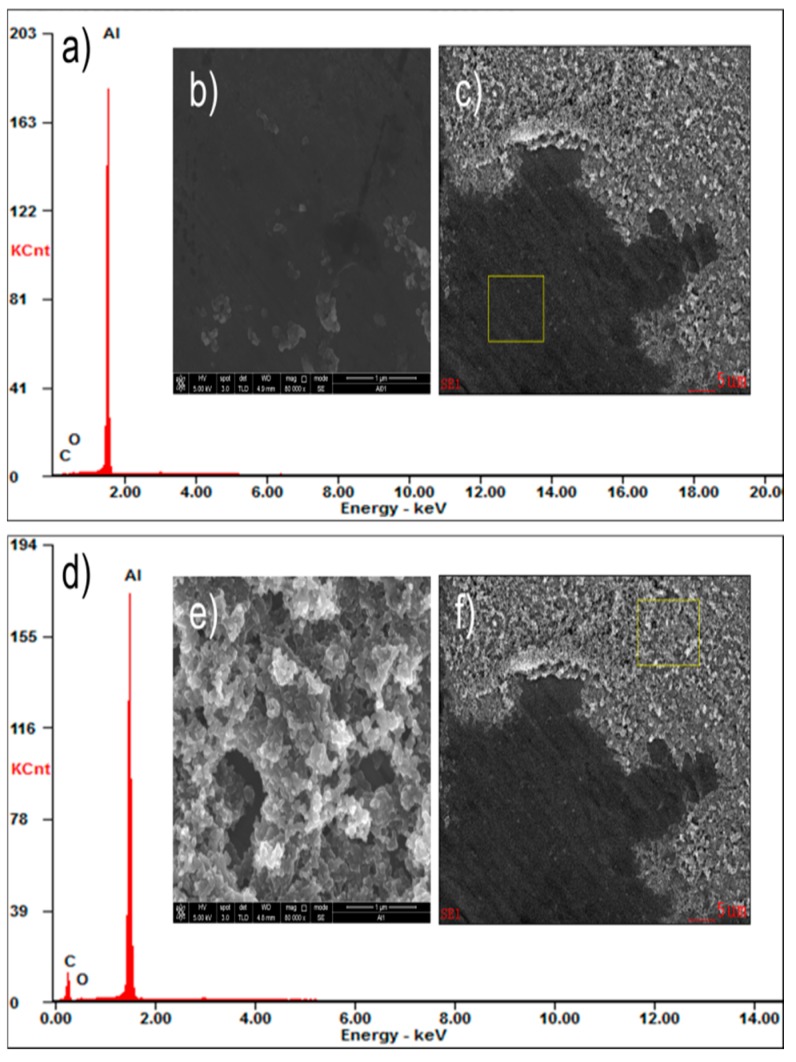
CSC-developed fingermark on an aluminum sheet characterized by (**a**,**d**) energy dispersion of X-rays (EDX) and (**b**,**c**,**e**,**f**) scanning electron microscopy (SEM). (**b**,**e**) High-resolution SEM image of the yellow rectangle regions.

A selection of the fingermarks on an aluminium sheet was examined by scanning electron microscope (SEM) for microstructure and microanalysis by EDX (energy dispersion of X-rays) to identify the reaction products. The SEM/EDX analysis was carried out and shown in Figure 2. It appears that the fingermark consists of compact layers of carbon soot and the EDX elemental analysis shows a higher carbon level in ridge regions (Figure 2a or Figure 2d). EDX analysis simply confirmed that carbon soot preferentially presented in the ridge area. Figure 2b is the magnified SEM image of a portion of aluminium substrates without CSC deposition shown in Figure 2c (marked with a yellow rectangle) while Figure 2e is the magnified SEM image of the area corresponding to the marked region covered by CSC in Figure 2f (marked with a yellow rectangle). Figure 2c or Figure 2f show a clear boundary between valley and ridge areas, indicating that carbon soot is spatially selectively coated. Moreover, the relatively stable structure of the carbon soot layer can keep the visualization of LFM for a long time.

**Figure 3:**
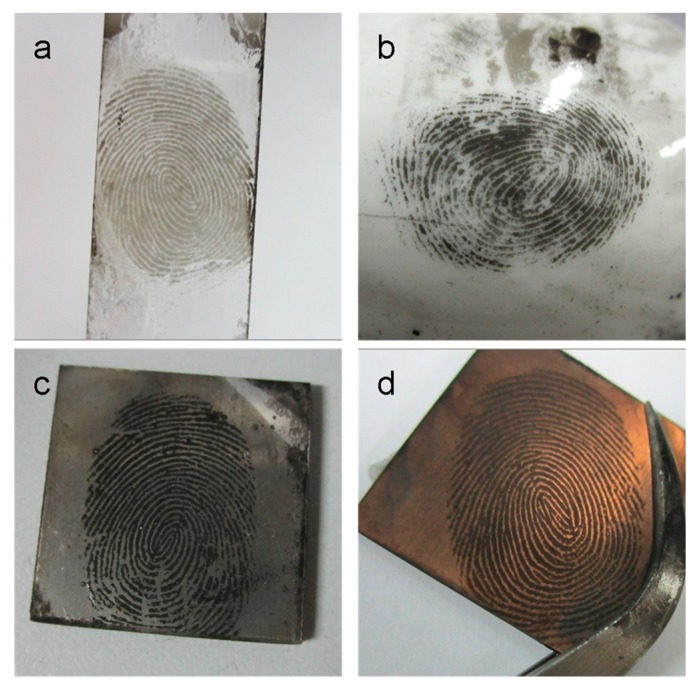

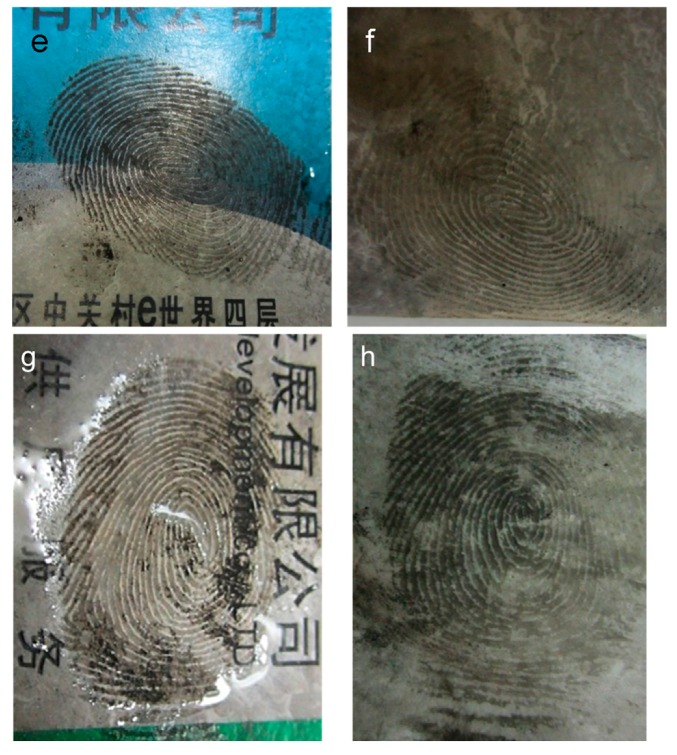
Fingermarks developed by the CSC method on several surfaces: (**a**) glass slide; (**b**) ceramic spoon; (**c**) aluminium sheet; (**d**) copper sheet; (**e**) cardboard; (**f**) adhesive tape; (**g**) paper card and (**h**) copy paper.

LFMs on various kinds of substrates which involve some porous/nonporous, conductive/insulative and smooth/rough surfaces have been further developed by CSC and the results are displayed in Figure 3. Figure 3a shows the LFM enhanced on a glass slide surface as a positive image. The second-level information such as the bifurcation, ridge termination, and crossover could be recognized distinctively. As for the LFM on a semi-porous ceramic material surface, the CSC technique was also able to enhance it with sufficient clarity to provide the second-level information shown in Figure 3b. The fingermarks on aluminum and copper materials which are commonly found on handled objects (door handles, bullets and etc.) have been well enhanced by CSC and the ideal fingermark image with second-level information shown in Figure 3c or Figure 3d, respectively. All the above results indicate that LFMs on the nonporous/semi-porous and conductive/insulative materials can be directly enhanced by using CSC without pre-treating the samples. In order to detect fingermarks on porous material surfaces such as paper, cardboard, etc., which are relevant in many crime scenes, LFM samples need to be pre-treated before CSC visualization to avoid sample damage due to its low ignition point. For example, LFM on cardboard which is often used in our daily life needs to be kept wet at first and then be developed by the CSC technique. Figure 3e exhibits the CSC-visualized fingermark on a normal cardboard surface which can also provide the second-level fingermark information. LFMs on paper card and copy paper can also be visualized by the CSC method (Figure 3g or Figure 3h). Therefore, it is proven that this CSC approach is capable of visualizing LFMs on a wide range of substrates.

At crime scenes, it is likely that fingermarks will need to be collected from most common or problematic/patterned substrates by adhesive tapes for further examination in a laboratory or in a fingerprint bureau; thus, we have further investigated a fingermark on adhesive tape and obtained a negative image in Figure 3f which is different from the positive images on other surfaces shown in Figure 3a–e. It is probably because the natural or synthetic glues used in adhesive tapes, based on acrylate, have a much stronger adherence to the carbon soot coating than the fingermark residue. A brush was used to remove redundant carbon soot instead of water flushing due to the soot being more difficult to remove from this adhesive surface. As can be seen in Figure 3f, the valley areas are much darker than the ridge areas. This negative fingermark image also has a similar resolution as the positive images developed on the other surfaces. The result shown in Figure 3f clearly demonstrates that adhesive tape was still in good condition.

Compared with other existing LFM enhancement approaches, such as VMD, MMD and ECD, CSC is simple, fast, low-cost and suitable for more kinds of substrates (porous/nonporous, conductive/insulative and smooth/rough surfaces).

## 3. Conclusions

In summary, we have demonstrated a simple and fast method for visualizing LFMs on various substrates by just burning a candle and soot deposition. In comparison with other deposition methods, CSC can rapidly visualize LFMs not only on various kinds of high temperature-resistant materials, but also on flammable surfaces, such as cardboard and copy paper. More importantly, it is also suitable for the enhancement of LFM on adhesive tape which is usually used for collecting fingermarks from many kinds of substrates at crime scenes. The developed fingermarks can be directly observed by the naked eye, without involving any sophisticated or expensive instruments for the enhancement.
